# Tiny timekeepers witnessing high-rate exhumation processes

**DOI:** 10.1038/s41598-018-20291-7

**Published:** 2018-02-02

**Authors:** Xin Zhong, Evangelos Moulas, Lucie Tajčmanová

**Affiliations:** 1 Earth Sciences Department, ETH Zurich, Sonneggstrasse 5, Zurich, 8092 Switzerland; 2 Present Address: Physics of Geological Processes, University of Oslo, Sem Sælands vei 24 Fysikkbygningen, Oslo, 0371 Norway; 3 Present Address: Institut des sciences de la Terre, Université de Lausanne, Inst. des sciences de la Terre Quartier UNIL-Mouline Bâtiment Géopolis, Lausanne, 1015 Switzerland

## Abstract

Tectonic forces and surface erosion lead to the exhumation of rocks from the Earth’s interior. Those rocks can be characterized by many variables including peak pressure and temperature, composition and exhumation duration. Among them, the duration of exhumation in different geological settings can vary by more than ten orders of magnitude (from hours to billion years). Constraining the duration is critical and often challenging in geological studies particularly for rapid magma ascent. Here, we show that the time information can be reconstructed using a simple combination of laser Raman spectroscopic data from mineral inclusions with mechanical solutions for viscous relaxation of the host. The application of our model to several representative geological settings yields best results for short events such as kimberlite magma ascent (less than ~4,500 hours) and a decompression lasting up to ~17 million years for high-pressure metamorphic rocks. This is the first precise time information obtained from direct microstructural observations applying a purely mechanical perspective. We show an unprecedented geological value of tiny mineral inclusions as timekeepers that contributes to a better understanding on the large-scale tectonic history and thus has significant implications for a new generation of geodynamic models.

## Introduction

The history of rocks is characterized by the pressure-temperature-time (*P-T-t*) path that can be reconstructed via interpretations of direct petrographic observations. Estimating the duration of exhumation often requires extracting a series of absolute ages by various radiometric-dating techniques which are often time demanding. Nonetheless, this temporal information is critical to construct the complete *P-T-t* path of the exhumed rocks.

**Figure 1:**
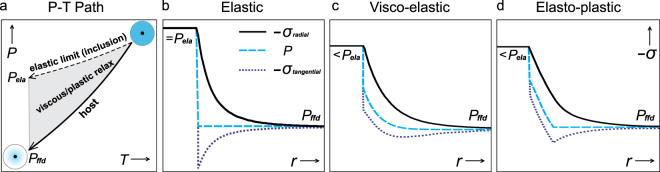
Exhumation *P-T* path and stress state in inclusion-host system. (**a**) The inclusion pressure (dashed) deviates from the host pressure (solid) with elastic rheology. It is termed the elastic limit *P*_*ela*_. The inclusion pressure is between the elastic limit and host pressure with viscous/plastic rheology. The far-field pressure is *P*_*ffd*_. (**b**) The inclusion pressure reaches the elastic limit with elastic rheology. The radial and tangential stress are *σ*_*radial*_, *σ*_*tangential*_. (**c**) The stress state of visco-elastic rheology. The inclusion pressure is lower than the elastic limit due to viscous relaxation. (**d**) The stress state of elasto-plastic rheology. The inclusion pressure is lower than the elastic limit due to plastic flow.

When rocks are exhumed, progressive cooling and decompression occur which results in a “pressure-vessel” effect on mineral inclusions (Fig. 1a)^1–4^. A pressure jump may develop between the inclusion and host as a result of their different bulk modulus and thermal expansivity. Assuming a spherical inclusion, the host adjusts to this pressure jump by spontaneously developing gradients of the radial and tangential stress components to achieve mechanical equilibrium (Fig. 1b). In exhumed rocks (particularly high-pressure/temperature rocks) experiencing cooling and decompression, such pressure jump may reach gigapascal (GPa) level as confirmed with the XRD and laser Raman spectroscopy^5,6^. It is known that minerals may undergo significant viscous creep or plastic yield when they are subject to differential stress of GPa level^7,8^. Viscous creep around inclusions is demonstrated by the stress induced dislocation structures observed with optical and transmission electron microscopy^9,10^. Plastic yield is manifested by radial/tangential cracks adjacent to inclusions^11,12^. Viscous creep continues as long as differential stress is present, implying that the amount of viscous relaxation of pressure jump is a function of time^13^. Therefore, the actual stress state in the host may not be perfectly elastic as shown in Fig. 1b. Instead, it may be time-dependent visco-elastic as in Fig. 1c^13,14^ or elasto-plastic in Fig. 1d^4^ (or visco-elasto-plastic). Although many studies using Raman spectroscopy reported residual pressure close to the predictions based on elastic model^6,15^, considerable amount of measurements of inclusions in a “crack-free” host show lower pressure than simulated with the elastic model, especially in high-pressure rocks^9,16,17^. It is very likely that viscous relaxation is responsible for these residual pressure measurements.

During exhumation, the ongoing development of the inclusion-host pressure jump due to the elastic effect and its continuous viscous relaxation operate simultaneously. The “clock” of the viscous relaxation starts ticking at the moment when exhumation begins from the Earth’s interior, and stops when temperature is sufficiently low to inhibit further viscous relaxation.

Here we show that high-pressure mineral inclusions in metamorphic and magmatic rocks can serve as important witnesses of the large-scale tectonic evolution through geological time based on a simple relationship between the residual inclusion pressure and the duration of exhumation. Such an alternative view of mineral inclusions offers a rheological chronometer based on the mechanical interaction between the inclusion and host, thus is entirely independent from the classical radiometric-dating techniques.

## Results

### Quartz/coesite in garnet

**Figure 2:**
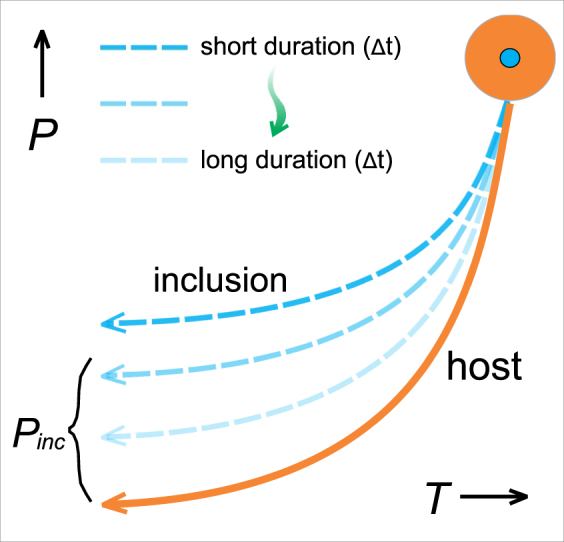
Relation between duration of exhumation (Δ*t*) and residual inclusion pressure (*P*_*inc*_). The orange curve is the host *P-T* path, and blue dash curves for inclusion. Lighter blue color corresponds to longer duration. With longer duration, lower residual inclusion pressure is produced due to viscous relaxation.

We focus on the commonly observed quartz/coesite-in-garnet system. The viscous flow-law for single garnet crystal has been experimentally measured for different garnet chemical compositions in e.g. Wang and Ji^18^ or Karato *et al*.^19^. We use finite difference method to compute the residual pressure of quartz inclusion corresponding to different duration of exhumation along discretized *P-T* paths. The Maxwell viscoelastic rheology is applied in numerical model, and temperature-dependent non-Newtonian viscosity is implemented (for more technical details about the numerical model see Methods). The numerical simulations yield a *P*_*inc*_*-*Δ*t* diagram (*P*_*inc*_ is the residual inclusion pressure after exhumation and Δ*t* the duration of exhumation) that allows us to recover Δ*t* given *P*_*inc*_ measured with Raman spectroscopy. A diagram in Fig. 2 shows a schematic correlation between *P*_*inc*_ and Δ*t*. A constant decompression rate is considered here which can be calculated from the peak pressure and duration. However, varying decompression rates can also be taken in the model by adding more variables describing the decompression history. In practice, this inclusion-host model involves many input parameters with uncertainties, and it is crucial to propagate these uncertainties into the *P*_*inc*_*-*Δ*t* relation. We consider four main sources of uncertainties: 1) the uncertainty of the experimental viscous creep data; the extrapolation of the viscous flow-law from the experimental to natural conditions; 2) the uncertainty of the retrograde *P-T* path; 3) the uncertainty of the entrapment *P-T* conditions for the inclusion; 4) the uncertainty of the measured residual pressure with Raman spectroscopy. These uncertainties are propagated into the calculated *P*_*inc*_*-*Δ*t* diagram with the analytical, bootstrap and Monte-Carlo methods (detailed in Methods). In our model, the inclusion is assumed to be a perfect sphere and the host is subject to isotropic stress. The final *P*_*inc*_*-*Δ*t* diagram can be envisaged as a mechanical counterpart of a radioactive half-life diagram used in radiometric-dating techniques, in that the *P*_*inc*_*-*Δ*t* diagram shows the “viscous decay” of the residual pressure as a function of time.

Two types of inclusion-host systems in different geological settings are chosen to test the model: 1) During a kimberlite eruption, a coesite-in-garnet system in a mantle xenolith is brought to the Earth’s surface within hours; 2) A quartz-in-garnet system is exhumed at a much slower rate in a metamorphic terrane. The duration of exhumation of these two scenarios vary by orders of magnitude, and often pose challenges to geochronological investigations (especially the rapid kimberlite magma ascent).

### Rapid kimberlite magma ascent

**Figure 3:**
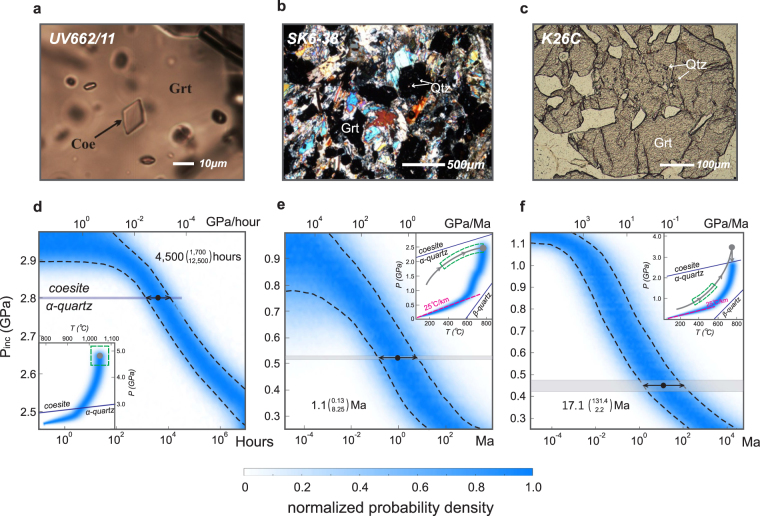
Selected microstructures and *P*_*inc*_*-*Δ*t* diagrams. Photomicrographs of: (**a**) Udachnaya kimberlite^20^; (**b**) Stak eclogite^17^; (**c**) Kulet whiteschist^16^ (figures taken from corresponding references). (**d–f**) *P*_*inc*_*-*Δ*t* diagrams following the same order as the upper panel. The colour shading characterizes the probability density of *P*_*inc*_. For *P-T* path, the colour shading characterizes the probability that the system stays at specific conditions. The green boxes show the possible entrapment *P-T* with equal probability inside. The horizontal shaded bands define the one standard deviation confidence interval for pressure measurements. The horizontal arrows and bracketed values characterize the one standard deviation confidence interval for the duration at mean pressure. The coesite-quartz transition is from Bohlen and Boettcher^32^, α−β quartz transition from Dorogokupets^49^.

Monocrystalline, euhedral coesite inclusions in garnet hosts have been found in the Udachnaya kimberlite (Russia) without having a transformed quartz rim (Fig. 3a)^20^. The host garnet grains are contained in mantle xenoliths with the origin at ~5 GPa, ~1,000 °C^20^. The explanation for the preservation of the high-pressure coesite at the Earth’s surface has been challenging. One possible explanation is the “pressure-vessel” phenomenon: the coesite inclusion is protected by the garnet host from dropping its pressure below the coesite-quartz transition boundary. However, the complete cooling and decompression of the garnet host from ~5 GPa, ~1,000 °C to room conditions will render the coesite’s overpressure lower than the coesite-quartz transition boundary based on the elastic model. Another explanation is that the coesite-quartz transition rate may be slow that inhibits the phase transition during rock decompression. This is not in agreement with the experiments demonstrating that the transition from coesite to quartz takes place in minutes at high-temperature (>800 °C)^21^. Therefore, it is very likely that during cooling and decompression, the coesite is first protected by the garnet host at high-temperature to maintain an overpressure, and only relaxes its pressure below the coesite-quartz transition boundary at low-temperature so that the transition is kinetically hindered. We calculate the duration along a segment of the constrained *P-T* path from 5 GPa, 1,000 °C to 2 GPa, 800 °C^20^. It is considered that 800 °C represents a sufficiently high temperature for the coesite-quartz transition. Above this temperature, the coesite-quartz transition occurs immediately. If pressure is below the coesite-quartz transition boundary and meanwhile temperature is above ~800 °C, a quartz outer rim should have been observed around the coesite inclusion. The calculated *P*_*inc*_*-*Δ*t* diagram in Fig. 3a shows that a very short period of ascent time ~4,500 hours is required to bring the coesite inclusion from the peak *P-T* conditions down to the phase transition boundary. This duration represents an upper limit for the magma ascent. A longer ascent duration (slower decompression rate) will cause the coesite pressure to decrease below the phase transition boundary, thus it is not in agreement with the petrographic observations (Fig. 3a). This case study shows that despite of viscous relaxation, high-pressure mineral inclusions can be preserved due to fast exhumation, in addition to the phase transition kinetic explanation. The calculated temporal bound (maximal ~4,500 hours) is valuable in that it allows to directly distinguish a catastrophic kimberlite magma ascent that is typically around hours or days^22,23^.

### Decompression of high-pressure metamorphic rocks

We constrain the duration of exhumation with the available Raman spectroscopy data for the two representative high-pressure rocks from the Stak eclogite in northwest Himalaya (Pakistan) and the Kulet whiteschist in the Kokchetav massif (Kazakhstan), where monocrystalline and round quartz inclusions are abundant in garnet crystals, thus providing suitable microstructural settings for our model.

The Stak massif is located in the Indus valley (northwest of Skardu, Pakistan) and has experienced the high-pressure event during the India-Asia continental collision^24,25^. The Stak eclogite follows a clockwise *P-T* path with the peak *P-T* at ~2.5 GPa, ~750 °C^26^, and went through ~1.3 GPa, ~610 °C conditions during exhumation^17,24^. The calculated *P*_*inc*_*-*Δ*t* diagram in Fig. 3b shows a maximal 1 GPa residual pressure if the duration is shorter than one thousand years. The measured residual inclusion pressure ~0.52 GPa (samples SK6-37 and SK6-38^17^) corresponds to ~1.1 Ma duration. Our results are similar to the duration reported in the geochronological studies in the nearby high-pressure rocks: (1) For Kaghan eclogite massif (~150 km away from the Stak massif), the duration of decompression is ~2.4 Ma from the peak conditions 2.75 GPa, 720~770 °C to <1 GPa, <600 °C conditions^27^; (2) For the Tso Morari eclogite massif (~400 km away), the duration of decompression is ~5.5 Ma from the peak conditions 2.55~2.75 GPa, 630~645 °C to 0.7~0.84 GPa, 705~755 °C conditions^28^. Our results provide an independent support for a rapid decompression rate (~2.3 GPa/Ma) of the Stak eclogite and its geodynamic relation with the nearby Kaghan and Tso Morari massif in the northwest Himalaya^17,25,26^.

In the Kokchetav massif, (ultra)-high-pressure metamorphic rocks that contain coesite and microdiamond inclusions were exhumed^29–31^. The peak *P-T* conditions of the whiteschist from the Kulet region are ~3.5 GPa, ~740 °C, which are followed by an isothermal decompression and subsequent cooling during the retrograde metamorphism^30^. The quartz inclusions entrapped in the garnet cores are observed to be monocrystalline and euhedral with no evidence showing coesite pseudomorph (bimineralic quartz-coesite and pure coesite inclusions are in the garnet mantle shown in Fig. 3c)^16,30^. This implies that the quartz inclusions in the garnet cores are protected by the host at a pressure below the coesite-quartz transition pressure, which is experimentally calibrated to be ~2.74 GPa at ~740 °C^32^. We perform the mechanical model following the retrograde *P-T* path constructed in Parkinson^30^ from the coesite-quartz transition ~2.74 GPa, ~740 °C to the surface conditions with an initial quartz inclusion pressure below 2.74 GPa (the uncertainty on the initial pressure detailed in Methods). The calculated *P*_*inc*_*-*Δ*t* diagram is shown in Fig. 3c. The duration of exhumation is constrained to be ~17.1 Ma based on the measured residual inclusion pressure ~0.44GPa^16^ (see Methods). This duration corresponds to an average decompression rate ~0.1 GPa/Ma. The calculated duration ~17.1 Ma for the Kulet whiteschist is in agreement with the geochronological results obtained by high-resolution ion microprobe dating on zircon in the Kokchetav massif: the dated duration of decompression and subsequent cooling from the peak *P-T* ~6 GPa, ~1,000 °C to amphibolite-facies conditions corresponds to <30Ma^33^.

## Discussions

The incorporation of viscous flow-law into the “pressure-vessel” model offers the first precise timing information based on microstructural observations and measurements from a pure mechanical perspective (*P*_*inc*_*-*Δ*t* diagram in analogy to the half-life diagram in radiometric-dating). The inclusion-host system can thus be used as a natural rheological chronometer that allows to: 1) quantitatively constrain the exhumation rate/duration along the retrograde *P-T* path with certain confidence interval, and 2) qualitatively distinguish the rocks originated from rapid magma ascent in e.g. volcanic eruption (time scale of hours) and slower metamorphic exhumation (time scale of Ma). We apply a constant decompression rate as a first-order approximation for the retrograde path. This approximation does not consider potential exhumation history with multiple stages because same inclusion residual pressure can be generated given different complex *P-T-t* path with varying decompression rate. However, in our numerical simulations, it is possible to apply a varying decompression rate through time. Such a model with complex *P-T-t* path can be further merged to large-scale geodynamic simulations to better deduce the tectonic evolution of a particular region. Given the current simple model with a constant decompression rate, the computed results better characterize the high-temperature part of the *P-T* path (near-isothermal decompression part), which experiences most of the viscous relaxation owing to the Arrhenius-type viscosity decay with respect to temperature increase. For a single garnet crystal maintained at temperature lower than 400~500 °C, the characteristic viscous relaxation time under GPa level shear stress may be even comparable to the age of the Earth (see Supplementary Information). Therefore, a “decelerating” decompression rate or long-term maintenance of the inclusion-host system at low-temperature may result in little change on the residual inclusion pressure.

The uncertainty for the constrained duration of exhumation is in exponential scale relative to the mean value as shown by the error-bars in the *P*_*inc*_*-*Δ*t* diagrams (Fig. 3). The results are more precise for rapid geological processes, e.g. the maximal duration for the Udachnaya kimberlite magma ascent is between 1,700 hours and 12,500 hours for one-sigma confidence level (Fig. 3a). Larger uncertainty is produced for longer duration of decompression, e.g. the duration for Kulet whiteschist is between 2.2 Ma and 131 Ma for one-sigma confidence level (Fig. 3c). Nonetheless, the lower bound (2.2 Ma) is useful as it places a lower limit for the duration of exhumation, thus is equivalent to an upper limit for the decompression rate (~1.3 GPa/Ma). Therefore, care needs to be taken when applied to slower exhumation lasting more than ten/hundred Ma due to the exponential uncertainty, e.g. Kulet whiteschist. However, the model is particularly accurate for fast magmatic/metamorphic processes where it might provide better time constraints than the classical radiometric dating methods with linear uncertainties on the absolute ages. Better experimental measurements on mineral/rock rheology and well-constrained *P-T* paths can improve the precision of the present model. Meanwhile, the uncertainty on residual inclusion pressure measurements is critical for reducing uncertainty of the determined exhumation duration. Therefore, (ultra) high-pressure rocks are particularly suitable for our model. This is because 1) the residual inclusion pressure is often large that gives smaller relative uncertainty on pressure measurement, and 2) the decompression duration is relatively short^34^, which results in smaller uncertainty of exhumation duration in exponential scale.

Microcracks (e.g. Fig. 1d) can significantly relax the inclusion residual pressure. Furthermore, palisade quartz involves large volumetric change that also deviates the residual pressure from the prediction of the visco-elastic model^4^. In order to apply the rheological chronometer, the quartz and coesite inclusions must be carefully examined so that they are crack- and palisade texture- free.

The rheological chronometer is established from a pure mechanical perspective to decipher the temporal information stored in the inclusion-host system. Such an alternative way is complementary to the conventional radiometric-dating data. The rheological chronometer, or more generally, the direct link from pressure measurements to geological time via viscous flow-law, can contribute to the study on high-rate exhumation processes where direct temporal data is often scarce.

## Methods

Here, we describe the modelling techniques for the inclusion-host systems, the methods for deriving the required physical parameters and the uncertainty propagation approaches. The figures and tables containing the relevant results are in the Supplementary Information.

### Mechanical formulations

We employ a Maxwell visco-elastic rheology with the deviatoric strain rate as the sum of the elastic and viscous counterparts (see e.g. Ranalli^7^). Due to the spherical symmetry, only the radial components of deviatoric stress/strain rate are treated.

1 $${\dot{e}}_{rr}={\dot{e}}_{rr}^{e}+{\dot{e}}_{rr}^{v}$$

where ${\dot{e}}_{rr}$ is the radial component of deviatoric strain rate in *s*^−1^ (the dot denotes the substantial time derivative). The superscripts *e*, *v* represent the elastic and viscous counterparts, respectively. The following constitutive relations are applied:

2 $${\dot{e}}_{rr}^{e}={\dot{\tau }}_{rr}/2G$$

3 $${\dot{e}}_{rr}^{v}={\tau }_{rr}/2\eta $$

where *G* is the shear modulus in *Pa*, and *τ*_*rr*_ is the radial component of the deviatoric stress in *Pa* (for simplicity, we refer *τ*_*rr*_ as deviatoric stress in the following text). The effective viscosity *η* (*Pa s*) is expressed as:

4 $$\eta =A{e}^{E/RT}{|{\tau }_{rr}|}^{1-n}$$

where *A* is the pre-factor in *Pa*^*n*^ *s*, *E* is the activation energy in (*J*)/(*mol*), *R* is the gas constant in (*J*)/(*Kmol*), *T* is the absolute temperature in *K*, and *n* is the stress exponent. The pressure dependence of viscosity is not considered here. Inserting the elastic and viscous deviatoric strain rate from equations (2) and (3) into equation (1), we get:

5 $${\dot{e}}_{rr}=\frac{{\dot{\tau }}_{rr}}{2G}+\frac{{\tau }_{rr}}{2\eta }$$

The stress rate is approximated with a first-order finite difference in time:$\,{\dot{\tau }}_{rr}\approx \frac{{\tau }_{rr}-{\tau }_{rr}^{o}}{{\rm{\Delta }}t}$, where ${\tau }_{rr}^{o}$ is the deviatoric stress from the previous time step. By doing so, equation (5) can be rearranged into the final visco-elastic constitutive relation in the current time step *τ*_*rr*_:

6 $${\tau }_{rr}=2\eta Z{\dot{e}}_{rr}+(1-Z){\tau }_{rr}^{o}$$

where $Z=\frac{G{\rm{\Delta }}t}{G{\rm{\Delta }}t+\eta }$ is the viscoelastic coefficient which approaches one for viscous rheology and zero for elastic rheology (see e.g. Gerya and Yuen^35^).

The conservation of mass writes:

7 $$\dot{\rho }+\rho \dot{\Theta }=0$$

where *ρ* is the density in $\frac{Kg}{{m}^{3}}$, $\dot{\Theta }$ is the volumetric strain rate in *s*^−1^, which is only induced by elastic deformation, i.e. no viscous volumetric strain rate is considered. The equation of state can be expressed as:

8 $$\frac{d\rho }{\rho }=\frac{1}{K}dP-\alpha \cdot dT$$

where *K* is the bulk modulus in *Pa*, α is the thermal expansion coefficient in *K*^−1^, and *P* is the pressure in *Pa* defined as positive for compression. Applying the time derivative on both sides of the above equation and inserting the result into equation (7) to eliminate $\dot{\rho }$, we get:

9 $$\dot{P}/K-\alpha \dot{T}+\dot{\Theta }=0$$

Approximating $\dot{P}$ and $\,\dot{T}$ with the first-order finite difference (same manner with ${\dot{\tau }}_{rr}$) and plugging the result into equation (9), we derive the pressure equation:

10 $$P={P}^{o}+K[-{\rm{\Delta }}t\dot{\Theta }+\alpha (T-{T}^{o})]$$

The momentum conservation equation neglecting the inertial term is expressed as follow:

11 $$\frac{\partial {\tau }_{rr}}{\partial r}+\frac{3{\tau }_{rr}}{r}-\frac{\partial P}{\partial r}=0$$

The volumetric strain rate and deviatoric strain rate in spherical coordinate can be explicitly written using the radial velocity *v*_*r*_ in *m*/*s*:

12 $$\dot{\Theta }=\frac{1}{{r}^{2}}\frac{\partial {r}^{2}{v}_{r}}{\partial r}$$

13 $${\dot{e}}_{rr}=\frac{\partial {v}_{r}}{\partial r}-\frac{1}{3}\dot{\Theta }$$

### Numerical techniques

The deviatoric stress and pressure in equations (6) and (10) is substituted into the momentum conservation equation (11) to get an explicit form containing the radial velocity *v*_*r*_. However, the explicit momentum conservation equation is nonlinear because the viscosity *η* is stress dependent. Therefore, direct iterations (Picard loop) are constructed to solve for the radial velocity *v*_*r*_, which can be used to compute for pressure and deviatoric stress. In the Picard iteration loop, the viscosity and viscoelastic coefficient are updated in each time step based on the calculated deviatoric stress. Meanwhile, the bulk modulus, shear modulus and thermal expansion coefficient are also updated during iterations. The boundary conditions apply for the far-field pressure and the overall temperature along the *P-T* path. The radial velocity at the inclusion centre (*r* = 0) is set as zero. The continuous *P-T* path is discretised into 100 segments for the numerical calculation. Here, we consider a constant decompression rate, thus each discretised *P-T* segment along the *P-T* path has equal-pressure and equal-time interval (total pressure and time divided by the number of segments). After the Picard iteration loop, the residuals of the momentum conservation equation are minimized to the machine precision. The material advection is not considered for this inclusion-host model as the displacement predicted is negligible. This model is benchmarked using the analytical solutions with elastic and non-Newtonian viscous rheology provided in the Supplementary Information (Supplementary Figs S1, S2).

### Fitting viscous flow-law

The viscous creep experiments from Wang and Ji^18^ are used here with only the ductile flow data (the brittle and semi-ductile experiments are not used). The flow-law used in their study is below:

14 $$\dot{\varepsilon }=B{(\frac{\sigma }{G})}^{n}exp(-g\frac{{T}_{m}}{T})$$

where *B*, *n* and *g* are the viscous creep parameters (*B* is in *s*^−1^ and *g*, *n* are dimensionless). A more convenient formula can be converted for data regression by applying a natural logarithm on both sides of the equation.

15 $${ln}(\dot{\varepsilon })-\,{ln}(B)-nln(\frac{\sigma }{G})+g\frac{{T}_{m}}{T}=0$$

The stress and temperature are individually scaled by the shear modulus and melting temperature (*T*_*m*_) for different garnet types (both are from Wang and Ji^18^). Different from the linear regression used in Wang and Ji^18^, we apply the generalized nonlinear inversion method to fit the three parameters ${ln}(B)$, *n* and *g*. Details of this approach can be found in Tarantola and Valette^36^ for the mathematical derivations, and Sotin and Poirier^37^ for the practical implementations and discussions particularly for the viscous flow-law. The regression results are summarized in the Supplementary Information (Supplementary Table S1). We set the experimental uncertainty for temperature as 10 °C, the natural logarithm of strain rate as 0.01, and the stress uncertainty as provided in Table 2 of Wang and Ji^18^ (ca. 10%). The pyrope-rich garnet is used here with shear modulus 98 GPa, and melting temperature 1,670 °C^19^.

In order to analyse the uncertainty associated with the experimental viscous flow-law, we apply the bootstrap approach (a statistical introduction of this approach has been provided in Efron and Tibshirani^38^). In garnet creep experiments, the temperature, strain rate and stress are measured in each run. With the bootstrap approach, each experimental run is considered as identically and independently distributed. The total 23 sets of the raw experimental data^18^ (denoted by ${D}_{i}=[{T}_{i},\,{\sigma }_{i},\,{\dot{\varepsilon }}_{i}]$) are resampled with replacement. A randomly generated example is shown below.

16 $${D}_{j}^{\ast }=\mathop{\underbrace{[{D}_{2},{D}_{16},{D}_{4},{D}_{2},{D}_{7},{D}_{9},{D}_{16},{D}_{4},\ldots \,\ldots {D}_{17}]}}\limits_{N=23}$$

The symbol with asterisk ${D}_{j}^{\ast }$ denotes the *j*th bootstrap data (containing 23 sets of experimental data). In this case, two sets of the raw data *D*_2_ and *D*_4_ are repeated twice due to the random replacement. Given this *j*th bootstrap data set ${D}_{j}^{\ast }$, the generalized nonlinear regression can be performed to fit the creep parameters ${ln}\,{(B)}_{j}^{\ast }$, ${n}_{j}^{\ast }$ and ${g}_{j}^{\ast }$. Such procedures are repeated more than 50,000 times (*j* > 50,000) to achieve a stable discrete probability distributions of ${ln}\,{(B)}_{j}^{\ast }$, ${n}_{j}^{\ast }$ and ${g}_{j}^{\ast }$, whose mean values converge towards the regressed creep parameters using the raw data without resampling. The inference of the creep parameters can be calculated using the discrete population distributions of the bootstrap results. The advantages of bootstrap approach in this specific case are: (1) The 3-by-3 variance-covariance matrix for the three creep parameters is implicitly taken into account by resampling from the experimental data. (2) The uncertainty of viscosity due to the creep experiments can be conveniently propagated by simply computing the effective viscosity for each bootstrap data set and analyse the discrete probability distribution of viscosity (this is particularly convenient for the uncertainty propagation of the numerical model explained in the next section).

### Propagation of uncertainties

The main uncertainties associated with the numerical computations come from: (1) The fitted viscous flow-law parameters, (2) the constrained *P-T* path, and (3) the entrapment conditions of the mineral inclusions. All the uncertainties must be fully respected and propagated into the numerical results from the afore-presented inclusion-host model.

In order to propagate the uncertainty of experimental viscous flow-law into the residual pressure, we use the bootstrap approach that has been introduced in the previous section. Here, it only takes one more step by applying the regressed creep parameters from the bootstrap data (${A}_{j}^{\ast }$, ${n}_{j}^{\ast }$ and ${E}_{j}^{\ast }$ converted from $\mathrm{ln}\,{(B)}_{j}^{\ast }$, ${n}_{j}^{\ast }$ and ${g}_{j}^{\ast }$) to the numerical model to calculate the final inclusion residual pressure. By doing so, the computed residual inclusion pressure carries the uncertainty related to the viscous flow-law extrapolated from the experimental conditions to the natural conditions. A stable discrete probability distribution of the residual inclusion pressure can be achieved given sufficient number of the bootstrap data.

The constrained *P-T* path based on petrological observations and thermodynamic modelling, taking the Kulet whiteschist from Parkinson^30^ as an example, is considered as the reference *P-T* path with the largest probability. The actual *P-T* path may, to some extent, deviate from this reference *P-T* path. Here, we define four evenly distributed control points (can be more than four depending on the shape of the *P-T* path) along the reference *P-T* path. We randomly deviate the *P-T* conditions around these four control points following the Gaussian distribution. Subsequently, we perform a second-order polynomial interpolation through the four randomly deviated *P-T* points to get a smooth *P-T* curve. It is also noted that near the Earth’s surface, the actual *P-T* path may simply follow the normal geothermal gradient without much deviations. Therefore, a *T*-controlled linear decay of the *P-T* deviations is implemented to narrow the scattering of the random *P-T* paths at low temperature. The peak temperature deviation (one-sigma standard deviation for the Gaussian probability distribution) used here is 25 °C, and the peak pressure deviation 0.1 GPa (for other samples they are also around 20~30 °C and 0.1~0.2 GPa). We show 10 possible *P-T* paths randomly generated for Kulet whiteschist using this approach in the Supplementary Information (Supplementary Fig. S4).

The entrapment *P-T* conditions of the quartz inclusion in the garnet host are often unknown. It is considered that the pressure of the inclusion and the host is the same when the inclusion is entrapped. The uncertainties of the numerical computations associated with the entrapment conditions are treated by choosing a segment along the prograde *P-T* path as the potential entrapment *P-T* range. Any entrapment *P-T* conditions within this *P-T* segment is equally probable. In each Monte-Carlo iteration, a random entrapment *P-T* point is chosen within this segment, and the inclusion pressure at the beginning of the modelled retrograde *P-T* path is computed using the elastic model. The application of the elastic model preserves the inclusion’s over(under)-pressure at the beginning of the retrograde *P-T* path, thus maximizing the uncertainty associated with the entrapment conditions. The uncertainties of the entrapment conditions are less significant (potentially negligible) if temperature is high or time duration is long. This is because significant viscous relaxation occurs at the very beginning of the numerical model to erase the initial inclusion’s over(under)-pressure.

To summarize, the procedures of the uncertainty propagation are: 1) Create the bootstrap creep data set with replacement from the raw experimental “data pool”. Perform generalized nonlinear inversion with the bootstrap creep data set to retrieve the creep parameters $\mathrm{ln}\,{(B)}_{j}^{\ast }$, ${n}_{j}^{\ast }$ and ${g}_{j}^{\ast }$, and convert them into ${A}_{j}^{\ast }$, ${n}_{j}^{\ast }$ and ${E}_{j}^{\ast }$. 2) Create a random Monte-Carlo *P-T* path with the Gaussian distributed deviations from the reference *P-T* path. 3) Compute the initial inclusion pressure with the elastic model by randomly choosing an entrapment *P-T* conditions prescribed within the *P-T* segment along the prograde *P-T* path. 4) Apply the fitted creep parameters (in Step 1), retrograde *P-T* path (in Step 2) and initial inclusion pressure (in Step 3) into the numerical model to compute the residual inclusion pressure after cooling and decompression. 5) Repeat from step 1 until a sufficient number of iterations is reached for a stable discrete probability distribution of the residual inclusion pressure. A pseudo-code is shown in the Supplementary Information to illustrate the work flow of the uncertainty propagation (Supplementary Fig. S5).

For one specific duration of exhumation, the above procedures generally take more than 50,000 iterations to get a statistically stable inference on the residual inclusion pressure. The duration is changed and the same procedures are repeated again in order to get the *P*_*inc*_*-*Δ*t* diagram. In total, more than 5 million bootstrap/Monte-Carlo numerical simulations are performed to get a statistically stable probability distribution. We use the high-performance cluster “*Euler*” in Lugano, Switzerland to perform this task with the access permission granted by ETH Zurich.

### Residual inclusion pressure with Raman spectroscopy

We apply the regressed experimental data from Schmidt and Ziemann^39^ to convert the measured frequency shift of the 464 cm^−1^ band into pressure for quartz inclusions. This applies for the sample of the Kulet whiteschist K26C (Table 3 in Korsakov *et al*.^16^). The 206 cm^−1^ band is not applied in sample K26C for the reasons related to the 206 m^−1^ band discussed in Ashley *et al*.^40^. For the Stak eclogite^17^, the same experimental calibrations from Schmidt and Ziemann^39^ are already used in the literature (sample SK6-37 and SK6-38). In this case, both 206 cm^−1^ and 464 cm^−1^ bands were used as they yield very consistent results^17^.

17 $$P(MPa)=0.3608{\rm{\Delta }}{{v}_{464}}^{2}+110.86{\rm{\Delta }}{v}_{464}$$

where Δ*v*_464_ is the frequency shift of 464 cm^−1^ band for quartz inclusion. The uncertainties of the Raman spectroscopy measurements can be introduced due to the limited spectral resolution. The uncertainty of the measured frequency shift is set as 0.05 cm^−1^ for the Stak eclogite^17,41^ and 0.2 cm^−1^ for the Kulet whiteschist^16^ (calibration protocol for error reduction following Hutsebaut *et al*.^42^). The uncertainty of the frequency shift can be analytically propagated into the residual pressure using the above equations.

### Elastic moduli

We use the modified Tait equation of state^43^ (EoS) for quartz and grossular, and the third-order Birch-Murnaghan EoS for almandine and pyrope in combination with the thermal pressure EoS^44^. The parameters for these EoS are from the database in the program EosFit7c^45^. The EoS parameters for spessartine is taken from Greaux and Yamada^46^ with a third-order Birch-Murnaghan EoS. The EoS parameters for coesite is from Duesterhoeft^47^ using the modified Bridgman power series. The average bulk modulus and thermal expansion coefficient for garnet is calculated from the composite volume based on a linear interpolation using the reported garnet compositions in the individual case studies. The shear modulus for garnet is from Bass^48^. Tests are performed to reproduce the same pressure-volume-temperature (*PVT*) relation for quartz, coesite and four garnet end members in the above references. All the above equations of state are programmed in a Matlab code that is incorporated into the inclusion-host numerical simulation. Readers are referred to EoSFit7c document^45^ for a summary of the equations of state, and the above cited references for the EoS parameters. The bulk modulus and thermal expansion coefficient of quartz are plotted in the Supplementary Information using the above-mentioned Tait EoS, and compared to the results calculated using the internally consistent thermodynamic database from Holland and Powell^44^ (Supplementary Fig. S6). The differences are not negligible. It is suggested to use the experimentally fitted EoS rather than the internally consistent thermodynamic database because the former is directly regressed using the *PVT* relationship measured experimentally.

## Electronic supplementary material

Supplementary Information
