# Supplementary material for: Tiny timekeepers witnessing high-rate exhumation processes
Source: Sci Rep. 2018 Feb 2;8:2234. doi: 10.1038/s41598-018-20291-7 (PMC5797167; doi:10.1038/s41598-018-20291-7)
Supplement: Supplementary file 1 — Supplementary Information [file 41598_2018_20291_MOESM1_ESM.pdf]

Supplementary Information

Tiny timekeepers witnessing high-rate exhumation processes

Zhong Xin[*1,2]*; Moulas Evangelos*[1,3]*; Tajčmanová Lucie*[1]

[1]*Earth Sciences Department, ETH Zurich, Zurich, Switzerland*

Now at: [2]*Physics of Geological Processes, University of Oslo, Norway*

Now at: [3]*Institut des sciences de la Terre, Université de Lausanne, Lausanne, Switzerland*

Contact email: xinzhong0708@gmail.com

Address: Earth Science Department, ETH Zürich, NO E 53.1, Sonneggstrasse 5, 8092, Zurich, Switzerland

This document contains the model benchmark for the inclusion-host system, and the supporting results, figures and table for the Methods section.

Model benchmarks

Elastic benchmark

Initially, the inclusion and host possess the same *P*-*T*. When the model starts, changes of far-field pressure and overall temperature are imposed on the inclusion-host system. The benchmark results are shown in Fig. S1. The normalized analytical (solid lines) and numerical (open circles) solutions of the stress and displacement are plotted as a function of the dimensionless radius (the analytical solutions are from Zhang[1]). Sufficiently large viscosity is prescribed to minimize

(eliminate) the effect of viscous stress relaxation. The displacement is numerically computed by integrating velocity with respect to time.

Non-Newtonian viscous benchmark

Initially, a higher pressure plateau in the inclusion, and a lower pressure sink in the host is pre-defined. The host material has different viscous stress exponent (n=1, 3, 5). When the model starts, the inclusion pressure gradually relaxes as a function of time. The analytical solution relating the inclusion pressure and time duration is provided in Dabrowski et al.[2], assuming that the host is purely viscous (incompressible and no elastic shear deformation). Here, such model setup is enforced by defining a five magnitudes larger bulk and shear moduli in the host compared to the inclusion. The viscous benchmark requires more than 3000 gridpoints in the radial coordinate, and the ratio between the host radius and the inclusion radius to be at least 100. Empirically, this is adequate to ensure that the numerical solution converges towards the analytical solution. The benchmark results are shown in Fig. S2.

Fitted viscous flow-law

The fitted parameters of the viscous flow-law based on the generalized nonlinear inverse method in this study and the ones based on linear regression in Wang and Ji[3] are shown in Table S1. The viscosity as a function of temperature at a fixed strain rate is plotted in Fig. S3 to compare the results in this study and Wang and Ji[3]. The results are very similar although the viscosity in Wang and Ji[3] is slightly higher than this study but is still within our 95% confidence interval. The inference of the viscosity is determined with the bootstrap approach detailed in Methods.

The uncertainty is smaller at the experimental conditions between 1,100 and 1,250°C and increases when the temperature is extrapolated to ~500°C.

The fitted creep parameters $ln(B)$ and g can be converted into A and E using Eq. 4 and Eq. 14 in Methods for the purpose of numerical modelling. Based on the calculation using the fitted parameters of viscous flow-law, the characteristic viscous relaxation time $t^* = \frac{2Ae^{E/RT}n^n(\Delta P_{ini})^{1-n}}{3(n-1)K_i}$ provided in Dabrowski et al.[2] is larger than ~1,000Ma at temperature <500°C for ΔP_{ini}=1GPa as the pressure difference between the inclusion and the far-field. This suggests that viscous relaxation can be negligible at temperature lower than 500°C for the quartz-in-garnet system.

Supplementary figures

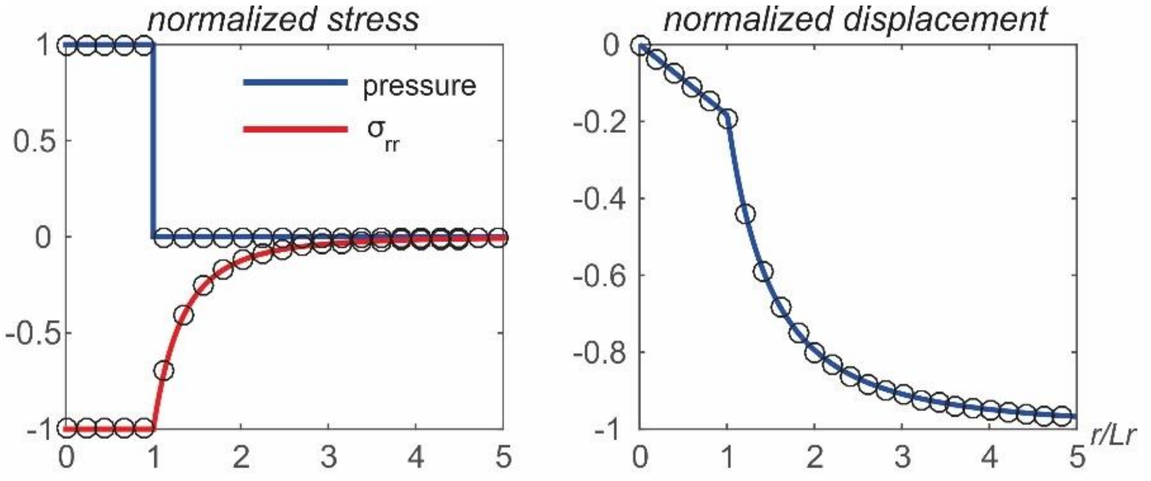

Figure S1 | Elastic benchmark. Analytical solutions are plotted as solid curves and numerical solutions plotted as open circles. Stress is normalized with the elastic limit (P_{ela}), and displacement is normalized by the far-field displacement.

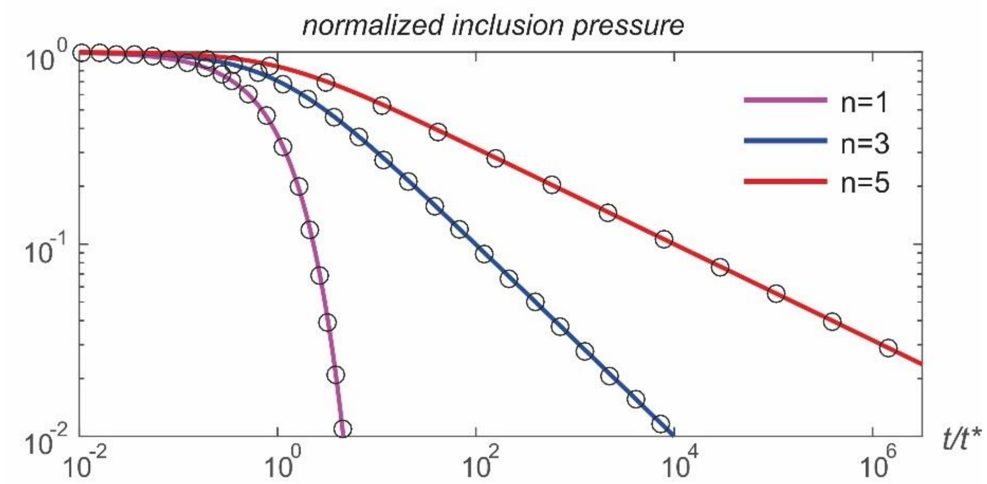

Figure S2 | Viscous benchmark. Analytical solutions are plotted as solid curves and numerical solutions plotted as open circles. The time scale $t^* = \frac{2Ae^{E/RT}n^n(\Delta P_{ini})^{1-n}}{3(n-1)K_i}$ for $n > 1$, and $t^* = \frac{2A_h}{3K_i}$ for $n = 1$,where ΔP_{ini} is initial pressure difference between the inclusion and host[2].

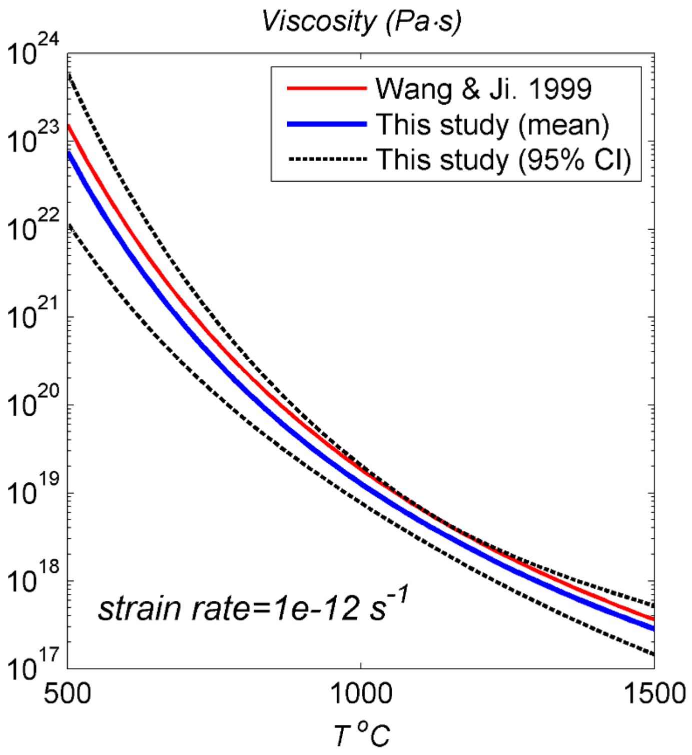

Figure S3 | Viscosity as a function of temperature. The red and blue curves are computed using the linearly regressed parameters from Wang and Ji[3] and this study with the generalized non-linear inversion method, respectively. The area bounded by the dash curves represents 95% confidence interval (CI).

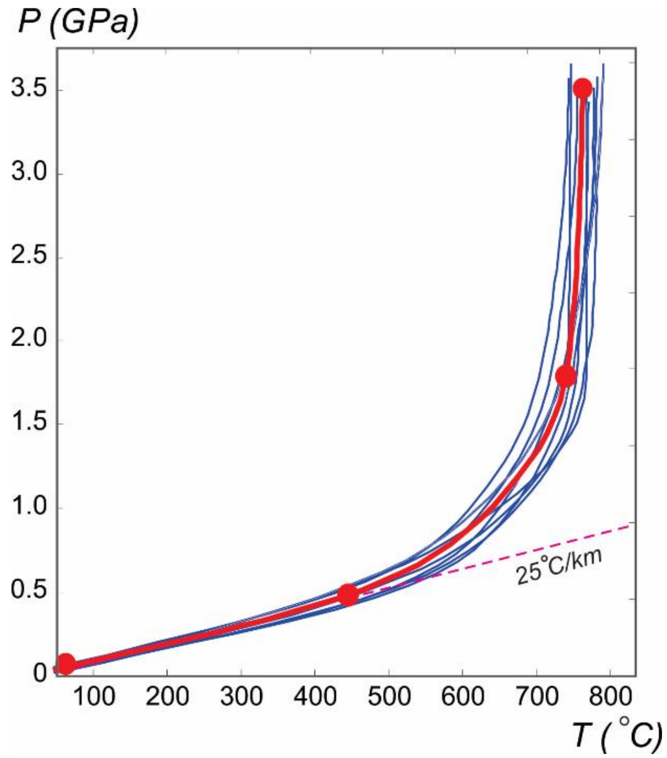

Figure S4 | Ten possible *P*-*T* paths generated randomly with Gaussian distributed deviations. The random *P*-*T* paths are located (blue curves) around the reference *P*-*T* path (red curve). The reference *P*-*T* path is from Parkinson[4]. At lower temperature, decreasing *P*-*T* deviations are prescribed along the geothermal gradient. The red dots are control points where the Gaussian distributed *P*-*T* deviations are implemented to generate each random *P*-*T* path.

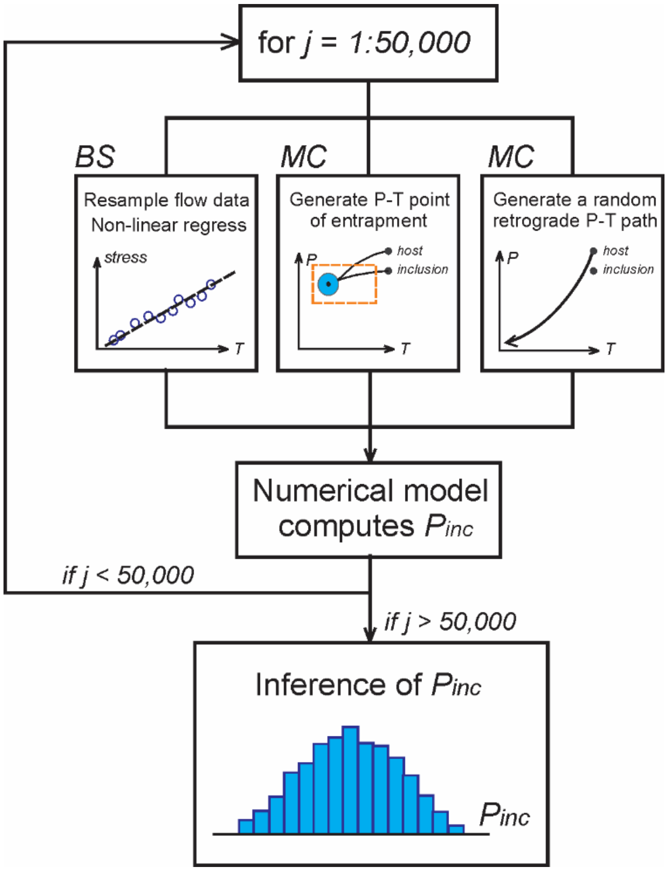

Figure S5 | Pseudo-code showing the uncertainty propagation procedures described in Methods. This schematic diagram shows how the uncertainties can be propagated from the experimental viscous flow-law, retrograde *P*-*T* path and entrapment *P*-*T* conditions into the final inclusion pressure. Iteration number for the bootstrap (BS) and Monte-Carlo (MC) is set at 50,000.

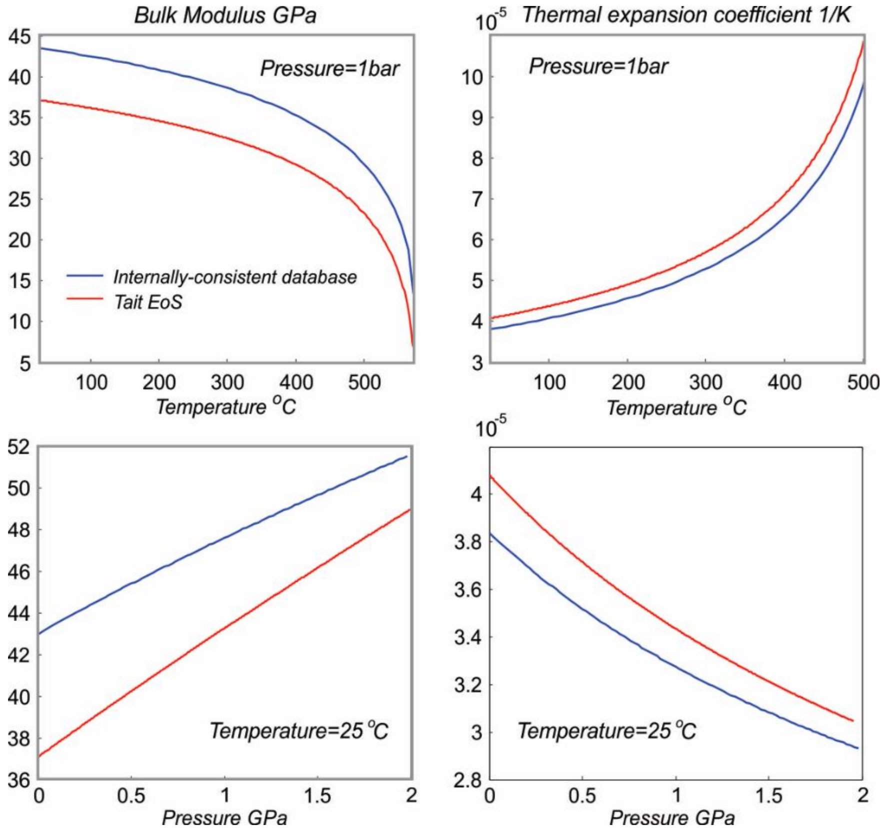

Figure S6 | Bulk modulus and thermal expansion coefficient of quartz. The blue curves are calculated using Perple_X[5] with the internally consistent thermodynamic database from Holland and Powell[6]. The red curves are calculated using the Tait EoS parameters from the program EoSFit7c[7]. Note the differences are not negligible.

Supplementary tables

Table S1| Regressed creep parameters in this study and Wang and Ji[3]. The uncertainty (plus-minus) characterizes one standard deviation of the fitted parameters.

	$ln(B)$	n	g
This study (generalized inversion)	36.68 ∓ 3.21	2.89 ∓ 0.14	29.41 ∓ 2.76
Wang and Ji[3] (linear regression)	40.1 ∓ 5.6	3.0 ∓ 0.5	32 ∓ 2

References

1. Zhang, Y. Mechanical and phase equilibria in inclusion-host systems. *Earth and Planetary Science Letters* **157,** 209–222 (1998).
2. Dabrowski, M., Powell, R. & Podladchikov, Y. Viscous relaxation of grain-scale pressure variations. *Journal of Metamorphic Geology* **33,** 859–868 (2015).
3. Wang, Z. & Ji, S. Deformation of silicate garnets: Brittle-ductile transition and its geological implications. *The Canadian Mineralogist* **37,** 525–541 (1999).
4. Parkinson, C. D. Coesite inclusions and prograde compositional zonation of garnet in whiteschist of the HP-UHPM Kokchetav massif, Kazakhstan: A record of progressive UHP metamorphism. *Lithos* **52,** 215–233 (2000).
5. Connolly, J. A. D. The geodynamic equation of state: What and how. *Geochemistry Geophysics Geosystems* **10,** Q10014 (2009).
6. Holland, T. J. B. & Powell, R. An improved and extended internally consistent thermodynamic dataset for phases of petrological interest, involving a new equation of

state for solids. *Journal of Metamorphic Geology* **29,** 333–383 (2011).

7. Angel, R. J., Gonzalez-Platas, J. & Alvaro, M. EosFit7c and a Fortran module (library) for equation of state calculations. *Zeitschrift fur Kristallographie* **229,** 405–419 (2014).
